# Supplementary material for: The Brain/MINDS Marmoset Connectivity Resource: An open-access platform for cellular-level tracing and tractography in the primate brain
Source: PLoS Biol. 2023 Jun 29;21(6):e3002158. doi: 10.1371/journal.pbio.3002158 (PMC10337976; doi:10.1371/journal.pbio.3002158)
Supplement: Attachment — Submitted filename: Response to Reviewers.pdf [file pbio.3002158.s001.pdf]

Response to Reviewers

We greatly appreciate the time and effort that the editor and reviewers have dedicated to reviewing our manuscript and providing us with valuable feedback for improvement. We have taken all reviewer comments into account and made revisions accordingly. In particular, we have rephrased lengthy sentences and paragraphs to enhance readability.

We would like to extend our sincere apologies for any inconvenience caused by the issue with the Nora StackApp. We have managed to reproduce the error on a Mac and have since resolved it. Additionally, we would like to highlight that the image data will be made available as open access nifti files. Upon acceptance of the manuscript, we plan to assign a DOI, share it with PLoS Biology, and update the Data Access section accordingly.

We have uploaded all the nifti files to the RIKEN CBS data portal (https://neurodata.riken.jp/mdrs/explorer/?id=39&path=%2FBMCR_v01%2F , folder BMCR_v01). Please note that the folder is currently password protected (password: BMCR_Marmoset_2023).

Please let me know if you need additional revisions or clarification. Thank you once again for your valuable input.

Responses to the Editor's comments

In addition, I would also be grateful if you could please address the following data and other policy-related requests that I have listed below (A-E):

(A)We would like to suggest that the following modification the title, to make it more accessible for our broad readership: "The Brain/MINDS Marmoset Connectivity Resource: an open access platform for cellular-level tracing and tractography in the primate brain"

We have replaced the title as suggested.

(B) In the Methods section of the manuscript, please provide additional details regarding housing conditions, feeding regimens, environmental enrichment, and all relevant steps taken to alleviate suffering of the animals (anesthesia, analgesia, details about humane endpoints, euthanasia, etc.). Also indicate how often animal care staff monitored the health and well-being of the animals and the criteria used to make such assessments. Lastly, specify the disposition of animals at the end of the study (e.g. euthanasia, returned to home colony, etc.). If animals were euthanized following the study, please provide the method of sacrifice.

We added a new section "Marmoset experiments" to the "Materials and Methods" section where we address all these points.

(C) You may be aware of the PLOS Data Policy, which requires that all data be made available without restriction: http://journals.plos.org/plosbiology/s/data-availability. For more information, please also see this editorial: http://dx.doi.org/10.1371/journal.pbio.1001797

Regardless of the method selected, please ensure that you provide the individual numerical values that underlie the summary data displayed in the following figure panels as they are essential for readers to assess your analysis and to reproduce it:

Figure 7A-C, 18A-D, 19D

For the figures 5, 7, 8, 18 and 19, we publicly share the python and Matlab scripts, as well as the data that we used to generate the plots and figures in the figure panels. We added notes to the figure captions and added further information to the "Data access" section. The code can be found here: (https://github.com/BrainImageAnalysis/BMCR/tree/main/BMCR_Figures). We share the image data and auxiliary files here: https://neurodata.riken.jp/mdrs/explorer/?id=39&path=%2FBMCR_v01%2F (folder "BMCR_v01", Password "BMCR_Marmoset_2023"). All passwords will be removed after publication.

*(D) Please also ensure that each of the relevant figure legends in your manuscript include information on *WHERE THE UNDERLYING DATA CAN BE FOUND*, and ensure your supplemental data file/s has a legend.*

Figures 3 and 4 are based on screenshots of the BMCR-Explorer and the Nora-StackApp. We added the information about which dataset was used for generating the figure legends. For the figures 5, 7, 8, 18 and 19, we mention in the figure legend where the Python and Matlab scripts and the data can be found.

(E) In line with the reviewer comments, please ensure that the tracer data is publicly available from the Brain/MINDS data portal and that the Nora StackApp is able to used by the user.

We uploaded all the nifti files to the RIKEN CBS data portal (https://neurodata.riken.jp/mdrs/explorer/?id=39&path=%2FBMCR_v01%2F, subfolder: "folder BMCR_v01/Img_data_tracer_Nissl_Backlit"). The folder is currently password protected (PW: "BMCR_Marmoset_2023"**). All data will be made publicly available without access restrictions after acceptance of the manuscript. We also plan to attach a DOI. After acceptance of the manuscript, we will also update all links on the Brain/MINDS data portal.**

Further, we found and fixed the issue with the Nora StackApp. We put installation and usage instructions on our web-page: https://bia.riken.jp/doku.php?id=tools:explorer#nora-stackapp and updated the portal page https://dataportal.brainminds.jp/marmoset-connectivity-atlas accordingly.

Any changes to the reference list should be mentioned in the cover letter that accompanies your revised manuscript.

The following preprint has been updated with a revised version:

Before: Watakabe et al. 2021 "Connectional architecture of the prefrontal cortex in the marmoset brain"

Revised: Watakabe et al. 2021 "Local and long-distance organization of prefrontal cortex circuits in the marmoset brain"

The following references were added:

- **Hata 2023, "Multi-modal brain magnetic resonance imaging database covering marmosets with a wide age range". Reason: Reference to detailed dMRI imaging protocols that we used to acquire the HARDI images.**
- **Cook 2007, "Optimal acquisition orders of diffusion-weighted MRI measurements". Reason: Reference to the gradient scheme used by the dMRI machine.**
- **Jones 1999, "Optimal strategies for measuring diffusion in anisotropic systems by magnetic resonance imaging". Reason: reference for the underlying methodology that we used to create the b-vectors of the HARDI template image.**

The following references were added to the section "Comparing anterograde neural tracer with dMRI tractography using BMCR data" to relate our findings to the state of art (please see our comments to reviewer #1 for details).

- **Maier-Hein 2017 "The challenge of mapping the human connectome based on diffusion tractography".**
- **Jeurissen 2019 "Diffusion MRI fiber tractography of the brain".**
- **Reveley 2015, "Superficial white matter fiber systems impede detection of long-range cortical connections in diffusion MR tractography".**
- **Donahue 2016, "Using diffusion tractography to predict cortical connection strength and distance: a quantitative comparison with tracers in the monkey".**
- **Liang 2023, "Using mesoscopic tract-tracing data to guide the estimation of fiber orientation distributions in the mouse brain from diffusion MRI".**

Responses to Reviewer #1

Reviewer #1: The authors present a multi-modal resource platform of the marmoset brain expected to be of broad interest in the community. Especially, the integration of both anterograde and retrograde tracers that have been reconstructed in 3D image volumes and the collection of ex vivo diffusion MRI on the same brain. The authors have constructed a 3D template of their multi-modal data and warped existing marmoset databases to their template increasing the data material and generality of usage across databases. Besides a comprehensive data processing

pipeline the authors also demonstrate a visualization tool. The data material and tool presented look highly convincing and of high quality.

(1) That said the manuscript could be more logically organized for the reader to follow such a complex setup and lacks comprehensive critical proofreading - too many sentences are too hard to follow.

We went through all sections and rephrased lengthy sentences that were hard to follow.

(2) The authors have selected to give "validation" examples of diffusion MRI-based tractography versus tracer's projections to demonstrate the usage of multimodality. It is basically a good idea and the authors do have unique data material including both anterograde and retrograde tracers. However, the examples used appear sloppy performed/unclearly presented. How are their validation findings related to the state of art validation and tractography issues?

We have revised the section "Comparing anterograde neural tracer with dMRI tractography using BMCR data" and related the work to the state of the art.

In summary, in non-human primates, neural tracer data from macaque brains have been used for qualitative or quantitative comparison with dMRI tractography data. However, available anterograde tracer data are sparse (Reveley, 2015), or not imaged alongside dMRI images , as in the case of retrograde tracers (Donahue, 2016). Others have used a mixture of hand-selected fiber tracts from real data and synthetic image data for dMRI validation (Maier-Hein, 2017), which might lack details of true connectivity beyond major fiber tracts. Our study marks the first time that a large dataset of anterograde tracer data from non-human primate brains has been made publicly available alongside high-quality dMRI measurements in the same image space. Anterograde tracers, in contrast to retrograde tracers, reveal true connection pathways. We believe that such data is essential for further studying how fiber tracking techniques and their parameters influence the resulting connectomes (Jeurissen, 2019 and Gutierrez, 2020). We also mention the potential importance of incorporating tracer data as anatomical prior to improve tractography techniques (Liang, 2023).

(3) Basically, the diffusion MRI modality data part needs to be tightened up. In the methods section experimental details are missing. How was the brain prepared for ex vivo MRI, how was it prepared in the MRI scanner, temperature drift of tissue that can impact diffusivity, argue why a b-value of 3000 s/mm^2 was chosen, how are the directions organized on the unit sphere, how long was the scan time, etc.?

(i) How was the brain prepared for ex vivo MRI and how was it prepared in the MRI scanner?

We added details regarding the preparation to the “The BMCR image data set” sub section in the “Materials and Methods” section. Animals were perfusion-fixed using 4% paraformaldehyde (PFA), and their brains were extracted for ex-vivo imaging. During this process, brains were encased in a sponge and submerged in a fluorine solution in a plastic container to prevent MRI interference. We added a reference to (Hata, 2022) which provides further details about the settings.

(ii) temperature drift of tissue that can impact diffusivity

We did not experience any temperature drifts. To ensure the best possible quality of our images, we carefully control the air conditioning at a temperature of 24 degrees Celsius and ensure that the probe is adequately warmed up to reach a steady state of tissue temperature. Additionally, we manually screen the results to ensure accuracy.
Further, before conducting diffusion measurements, we typically take T2 and low-bvalue measurements for approximately 5 hours. This allows for sufficient time to reach a steady state, which is crucial for obtaining reliable results.

(iii) argue why a b-value of 3000 s/mm^2 was chosen

In the manuscript, we inadvertently omitted information regarding additional measurements. Specifically, we conducted measurements using b-values of 1000, 3000, and 5000 s/mm^2. We have now added this information to the manuscript in the "The BMCR image data set" and "Pipeline inputs" sections. These values were selected to account for the lower diffusivities observed in ex-vivo tissue samples, which were approximately 2.5 times smaller than those in postmortem specimens. This choice of b-values allowed us to achieve similar sensitivities and contrast-to-noise ratio (CNR) relationships as the in-vivo scheme that facilitates "standard white matter" modeling with multi-compartment models, as described by Novikov (2018). The in-vivo scheme typically consists of b-values 0, 1000, and 2000; therefore, we adjusted our ex-vivo b-values by multiplying them by approximately 2.5, resulting in our proposed scheme of b=0, 1000, 3000, and 5000.

(iv) how are the directions organized on the unit sphere

We image with the Bruker's standard recommended settings (equidistantly distributed points on a sphere). Bruker provided us the corresponding reference which we have added to the manuscript (Cook at al 2007).

(v) how long was the scan time

3 h, 20 min for the T2 image, and 6 h, 39 min for the dMRI image. We added this information to the *"The BMCR image data set" section.*

(4) The ex vivo setup lacks references to relevant literature.

We added a reference with further details about the dMRI acquisition method (Hata 2023).

(5) Further, the terminology used for tractography is confusing and can be misunderstood when compared with tracer data. Suggest avoiding using "Fiber" in terms of tractography but using "streamlines" and "streamline density" instead of "fiber density". Fiber density can be misunderstood as related to axon density, but the number of streamlines is not related to axon density but more to how many streamlines emanate from a seeding region.

Thanks for the pointer. We replaced the terms accordingly, including their occurrence in Fig. 5.

(6) Generally, the method sections could be clearer. The discussion includes "Originality and significance" but not clear why it is needed. Overall, maybe the authors should consider simplifying the manuscript so the reader can appreciate the interesting multi-modal resource they wish to provide.

Thanks for the suggestion. Indeed, the section "Originality and significance" is quite repetitive, short and the cited works have already been mentioned in the introduction. We have decided to delete the "Originality and significance" section. Further, we went through all sections and rephrased lengthy sentences that were hard to follow.

Responses to Reviewer #2

Reviewer #2 (Stefan Everling, signs review): Skibbe and colleagues present here an open access platform for anterograde and some retrograde tracer data for different prefrontal areas in the marmoset. This is a fantastic resource! I have already played around with the BMCR viewer for hours and this dataset will be an extremely valuable resource for anyone working in marmoset neuroscience or who is interested in aspects comparative neuranatomy. I really would like to thank the authors for collecting these data and for putting this very impressive resource together. I cannot comment on the fine details of the actual processing pipeline but the results look amazing and seem to be of very high quality. I only have a few comments.

Major comments
1. The BMCR explorer is easy and pretty intuitive to use. The functionality is truely impressive! However, I could not get the NoraStack app to work at all and therefore could not evaluate it.

We could replicate the problem on a Mac (M1). There was a bug that has been fixed.

2. I also could not download the actual tracer data from the website. Are they actually now open access? I think they should be so that researchers can analyze them with their own analysis tools. The current download folder is password protected and none of the supplied passwords worked.

We uploaded all the nifti files to the RIKEN CBS data portal (https://neurodata.riken.jp/mdrs/explorer/?id=39&path=%2FBMCR_v01%2F, subfolder: " BMCR_v01/Img_data_tracer_Nissl_Backlit"). The data include the following nifti files:

- **Raw anterograde tracer signal (3 channels)**
- **segmented tracer signal**
- **tracer density images**
- **Injection site cell density images**
- **Nissl and Backlit images**

We further provide:

- **The locations of detected cells in each injection site**
- **The center of the injection site**
- **dMRI fiber tracks that are associated with the injection site**

The folder is currently password protected (PW: "BMCR_Marmoset_2023"). All data will be made publicly available without access restrictions after acceptance of the manuscript. We also plan to attach a DOI. An overview of available files is listed in Table 1 in the manuscript.

3. The authors should really include the (lower resolution) tracer data as nifti files so that they can easily be combined with other imaging data such as MRI and fMRI by other groups. Basically similar to what is already included in marmoset brain connectivity atlas (marmosetbrain.org). The BMCR viewer is really nice but having the data also in nifti format would be extremely useful.

The image data can be downloaded in nifti format from the RIKEN CBS data portal (see our response to point two). Links on the Brain/MINDS landing page will be updated once the manuscript is accepted.

Minor comments

P3,line 26-27: the authors could also mention the granular prefrontal cortex of primates here
P11, line 197 "generated"
P16, line 367: replace "was" with "of"
P17, line 399: "lunch" should be "launch"

Lines 503-505, figure: change to "sagittal"

Thank you very much for the pointers. We have corrected the typos.

Responses to Reviewer #3

Reviewer #3 (Matthew F. Glasser, signs review): The authors present what is a super impressive body of work on invasive and non-invasive marmoset connectivity. I found the software somewhat more frustrating (had some success with the BMCR-Explorer but could not get the Noraview to work on Mac with multiple error messages when attempting to load files or folders). I saw that both software had a concept of a "scene" where one saves the state of the software. That could have been used to better advantage to hold the hand of the novice user, as the readme for Noraview was extremely terse. My main suggestion would be to get some colleagues unfamiliar with the tools to try them out and give notes on what works well and what doesn't.

We could reproduce the problem with the Nora app on Mac, found a bug, and fixed it. As suggested, we asked a person unfamiliar with the tool to download it, try it out, and take notes. Based on the notes, we put instructions on our web-page: https://bia.riken.jp/doku.php?id=tools:explorer#nora-stackapp. We also updated the portal page https://dataportal.brainminds.jp/marmoset-connectivity-atlas accordingly.

It could be a little clearer that the diffusion data were from multiple animals (in some places this is clear, others less so).

Indeed, the introduction was a bit ambiguous about that part. Thank you very much for the pointer. In the revised manuscript we clearly say that we share the registered dMRI images from 23 animals.

It was unclear to me why the diffusion data and templates in general needed to be symmetrized. Perhaps this could be justified.

The template is created from the autofluorescent signal of the brain tissue. Strong tracer signals, however, can leak into the recordings of the autofluorescent signal. We chose a symmetric template because all injections were placed in the left hemisphere. Averaging the left and right hemispheres allowed us to double the number of samples and minimize potential biases. Additionally, the symmetric template streamlined the annotation of brain structures and facilitated the integration with the Marmoset Brain Connectivity Atlas, which provides data for only one hemisphere. We added this information to the manuscript (Section "The processing pipeline")
